# Numerical and Thermal Response of the Bacterivorous Ciliate *Colpidium kleini*, a Species Potentially at Risk of Extinction by Rising Water Temperatures

**DOI:** 10.1007/s00248-024-02406-y

**Published:** 2024-07-02

**Authors:** Thomas Weisse, Thomas Pröschold, Barbara Kammerlander, Bettina Sonntag, Laura Schicker

**Affiliations:** 1 Research Department for Limnology, University of Innsbruck, Mondsee, Austria; 2 Department of Ecology, University of Innsbruck, Innsbruck, Austria; 3 Present Address: Federal Agency for Water Management, Institute for Aquatic Ecology and Fisheries Management, Mondsee, Austria

**Keywords:** Ciliates, *Colpidium kleini*, Growth rates, Mortality rates, Numerical response, Thermal performance curves

## Abstract

We investigated the food-dependent growth and thermal response of the freshwater ciliate *Colpidium kleini* using numerical response (NR) experiments. This bacterivorous ciliate occurs in lotic water and the pelagial of lakes and ponds. The *C. kleini* strain used in this work was isolated from a small alpine lake and identified by combining detailed morphological inspections with molecular phylogeny. Specific growth rates (*r*_*max*_) were measured from 5 to 21 °C. The ciliate did not survive at 22 °C. The threshold bacterial food levels (0.3 − 2.2 × 10^6^ bacterial cells mL^−1^) matched the bacterial abundance in the alpine lake from which *C. kleini* was isolated. The food threshold was notably lower than previously reported for *C. kleini* and two other *Colpidium* species. The threshold was similar to levels reported for oligotrich and choreotrich ciliates if expressed in terms of bacterial biomass (0.05 − 0.43 mg C L^−1^). From the NR results, we calculated physiological mortality rates at zero food concentration. The mean mortality (0.55 ± 0.17 d^−1^) of *C. kleini* was close to the mean estimate obtained for other planktonic ciliates that do not encyst. We used the data obtained by the NR experiments to fit a thermal performance curve (TPC). The TPC yielded a temperature optimum at 17.3 °C for *C. kleini*, a maximum upper thermal tolerance limit of 21.9 °C, and a thermal safety margin of 4.6 °C. We demonstrated that combining NR with TPC analysis is a powerful tool to predict better a species’ fitness in response to temperature and food.

**Supplementary Information:**

The online version contains supplementary material available at 10.1007/s00248-024-02406-y.

## Introduction

Protist ecology has increasingly shifted from a taxonomic-oriented approach towards an ataxonomic, trait-based functional approach over the past decades [1–3]. The main reason is that key processes in aquatic and terrestrial ecosystems usually depend on the functional performance of groups of similar organisms. Functional ecology seeks to identify and parameterize critical processes such as production, consumption, and remineralization rates in the ecosystem context. Similar to their colleagues working with macroorganisms, experimentally working protistologists increasingly focus on studying model organisms [4–7] that represent major functional traits in the ecosystem.

Several organismic traits (e.g., specific growth rates, production rates, threshold resource levels needed to sustain a population) are linked to resource consumption. Resource uptake can be generally characterized by a rectangular hyperbolic function similar to Michaelis–Menten kinetics [4, 5]. For phagotrophic organisms, the numerical response (NR) portrays the performance of a predator or grazer as a nonlinear function of resource availability (food or prey [6, 7]). Numerical response experiments, usually conducted in the laboratory, estimate specific growth rates in response to biotic (food supply) and abiotic variables (e.g., temperature, pH, and salinity [8]). The parameter estimates of the NR curve (see Eq. (2), Materials and Methods) can be used in mathematical models of ecosystem dynamics. Such trait-based models are, for instance, needed to predict how planktonic organisms will adapt to global warming and how this adaptation will impact the pelagic carbon flux [9].

Like NR curves, thermal performance curves (TPCs) provide several significant trait-based functions. Notably, TPCs are instrumental in predicting the organisms’ fitness to environmental temperature changes [10–12]. Two of the three parameters characterizing the TPC, namely the temperature optimum (*T*_*opt*_) and the critical upper thermal tolerance limit (*CT*_*max*_), are crucial for predicting species’ survival in a warmer world. The difference between *CT*_*max*_ and *T*_*opt*_ defines the thermal safety margin (TSM, [13, 14]) that predicts the species’ survival at supraoptimal temperatures. The critical lower temperature tolerance limit (*CT*_*min*_) describes the minimum temperature needed to survive.

Ciliates (Ciliophora) are a phylogenetically well-characterized, species-rich (likely > 20,000 species, [15]), and physiologically highly variable protist phylum [16]. Ciliates are found in virtually every terrestrial and aquatic environment and play a pivotal role in planktonic food webs (reviewed by [9]). Food web models demonstrated the significance of planktonic ciliates for the pelagic carbon flow [17, 18] and predicted their response to global warming [19].

Numerical response experiments with ciliates are available from 41 published studies investigating 56 species or strains in 81 NR experiments [20]. Most NR experiments dealt with algivorous or omnivorous ciliates; NR curves are only available for a handful of bacterivorous ciliate species. Similarly, the evidence on the thermal response of aquatic ciliates is limited; TPCs are available for 25 ciliate species, comprising two marine and 23 freshwater taxa [21, 22]. Much of the previous work focused on model ciliates of the genera *Tetrahymena* (seven species) and *Paramecium* (three species), whereas common planktonic species received little attention.

Numerical responses and TPCs were explored independently, although several studies reported ciliate NR at different temperatures (compiled in [20]). The TPCs provide an alternative estimate of the specific growth rate (*r*_*max*_). We will demonstrate that combining both methods is a powerful tool for better predicting a species’ fitness in response to temperature. We assumed that NR curves would overestimate *r*_*max*_ because the rectangular hyperbolic model often predicts *r*_*max*_ at food levels beyond those studied and commonly encountered in situ (see below Discussion). Therefore, we hypothesized that both methods yield different estimates of *r*_*max*_ under realistic natural conditions.

We also tested the effect of two modifications of NR curves on the estimated model parameters. To this end, we related the ciliate’s growth rate to either (*i*) the initial food abundance or (*ii*) the mean food biomass during the experiments. Our null hypothesis was that the different statistical treatments would not affect the results obtained.

We conducted our experimental study with the bacterivorous freshwater ciliate *Colpidium kleini*, first described by Foissner [23]. Since unequivocal identification of this species is difficult and its taxonomic affiliation has been debated [24], we combined comprehensive morphological inspections with SSU and ITS rDNA sequence analyses. We compared our experimental results to previous work on two other bacterivorous *Colpidium* species [25] and other ciliates. We discussed the implications of our study for future research on the numerical and thermal responses of phagotrophic protists.

The *C. kleini* strain used in this study was isolated from a small alpine lake in Austria. Such pristine lakes are particularly susceptible to temperature change [26]. Since *C. kleini* did not survive at 22 °C, we conclude that this ciliate may be at risk of extinction in temperate small and shallow water bodies if global warming continues.

## Materials and Methods

### Sampling Site, Study Organisms, and Experimental Design

*Colpidium kleini* Foissner, 1969 was sampled from the pelagial of Lake Rifflsee, Austria, on Aug 23, 2010, by vertical net hauls using a 10-µm plankton net. The alpine Rifflsee (located at 2,234 m a.s.l., surface area 0.269 km^2^, max. water depth 24 m) is characterized by high turbidity caused by glacial inflow. The average water temperature is close to 8 °C, and the maximum is ~ 12 °C [27, 28]. The bacterial abundance, measured in 2% formalin-fixed material after DAPI staining (see [29] for details), ranged from 7.6 × 10^5^ to 1.1 × 10^6^ cells mL^−1^, i.e., it was comparable to bacterial levels of similar small lakes in the western Austrian Central Alps [29]. Chlorophyll *a* was below 1 µg L^−1^ in the summer of 2010 [27].

*Colpidium kleini* is not euplanktonic and is more common in small water bodies (ponds and wastewater treatment plants [30, 31]) than in larger lakes. It is often associated with macrophytes [30]. The species does not form resting cysts [30]. Thus far, *C. kleini* has been reliably reported only from temperate Europe, although its occurrence in North America is likely ([30, 31] and references therein).

After sampling, individual ciliate cells were cleaned, cloned, and cultivated as described in [28]. Clonal cultures were kept non-axenically in an enrichment culture with a modified WC medium ([32]) containing algae (*Cryptomonas* sp. strain SAG 26.80, http://sagdb.uni-goettingen.de/detailedList.php?str_number=26.80) at low temperature (^~^ 5 °C) in the dark. The clonal strain used in this study was named CIL-4.

The clonal cultures were adapted to a bacterial diet devoid of algae by transferring individual cells to 24-well tissue culture plates and feeding the ciliate with the heat-killed γ-Proteobacterium *Listonella pelagia* strain CB5 (Gen-Bank synonym *Vibrio pelagius* [33]). The ciliate culture volume was gradually increased to 10 mL (in 6-well plates) and culture flasks of 50 mL volume (stock cultures). Stock cultures were maintained in the dark at 10 °C.

The numerical response experiments were conducted in 6-well plates in the dark over 24 h from autumn 2022 to early 2023. The ciliates were first stepwise adapted to four temperatures (5, 10, 15, and 20 °C). Depending on the temperature, the ciliates were then acclimated to the final experimental conditions for 1 − 3 days. Experimental temperature ranged from 5 to 21 °C. The highest two temperatures (i.e., 19 °C and 21 °C) were chosen to measure the ciliate’s performance close to its optimum temperature (*T*_*opt*_) and upper-temperature tolerance limit (*CT*_*max*_). Our attempts to acclimatize the ciliate to higher temperatures failed. Each temperature represented a treatment. A treatment consisted of 24 experimental containers of 10-mL volume, each with varying food levels. Initial prey densities ranged from approximately 0.1–10 × 10^6^ bacteria mL^−1^, and initial ciliate abundance from 11–63 cells mL^−1^ (20–40 cells mL^−1^ in most experiments). Following earlier recommendations [6], we did not use replicates but spread our measurements across the food range and conducted more experiments at lower bacterial concentrations than at supposedly satiating food levels. Therefore, each food level represents a single experiment of a temperature treatment. This method yields more accurate parameter estimates for nonlinear curve fitting than replicating standard food levels [6].

### Analyses

Subsamples for measuring ciliate and bacterial abundances (1 mL for bacteria, 3 mL for ciliates) were taken at the beginning and end of each experiment. Ciliate abundance and cell size were determined from acid Lugol’s fixed material (2% final concentration, vol/vol). Cell length and width were measured in three of the five treatments (at 10 °C, 15 °C, and 19 °C) by an image analysis system (NIS elements D; Nikon CEE GmbH) connected to an inverted microscope (Zeiss Axiovert 200). Cell volume was estimated assuming the shape of a prolate spheroid [34].

Bacterial abundance was measured from formalin-fixed material (2% final concentration) using an AttuneTM NxT Acoustic Focusing Cytometer (Life Technologies, Austria, Thermo Fisher Scientific Inc., Vienna, Austria) following [35]. Bacterial cell size was determined by epifluorescence microscopy (Olympus BX53, Olympus, Vienna, Austria) and image analysis (NIS-Elements D3.2. 64-bit) at 1,250 × magnification after DAPI staining [36]. Bacteria were counted and sized as cocci or small rods, and the cell volumes were calculated assuming appropriate geometric shapes.

Ciliate growth rates were estimated from the change in cell numbers during the experiments assuming exponential growth:

1 $$r = \text{ln }({N}_{t}/{N}_{0})/t$$

where *N*_*0*_ denotes the initial, and *N*_*t*_ is the final ciliate abundance (cells mL^−1^); *t* is the experimental duration (d).

The numerical response is calculated from Eq. (2):

2 $$r = {r}_{max} (P- P{\prime}) / [k + (P- P{\prime})]$$

*r*_*max*_ is the maximum specific growth rate (d^−1^), *P* is the prey level during the experiments, *k* is a constant (cells mL^−1^), and *P'* is the x-axis intercept (i.e., the threshold concentration, where *r* = 0). The data (i.e., *r* vs. *P*) were fitted using nonlinear curve fitting with SigmaPlot for Windows (version 14.5.0.101).

Prey levels were estimated (*i*) as initial bacterial abundance (cells mL^−1^) and (*ii*) as geometric mean bacterial biomass (mg C L^−1^) during the experiments. For the latter, mean bacterial abundance was converted to carbon biomass assuming the measured cell volumes of 0.14 µm^3^ (cocci), respectively 1.25 µm^3^ (rods), and a conversion factor of 200 fg C µm^−3^ [37]. The geometric mean prey concentration (*P*) was determined as follows:

3 $$P = ({P}_{t} - {P}_{0}) /\text{ ln }({P}_{t} - {P}_{0})$$

*P*_*0*_ and *P*_*t*_ are the initial and final food concentrations (cells mL^−1^).

Maximum mortality rates (*δ*_*max*_, d^−1^) were calculated from Eq. 2, setting *P* to zero [38]. *δ*_*max*_, also known as physiological mortality or physiological death rate, is the counterpart of *r*_*max*_ achieved under optimal, food-replete conditions [20].

To compare our results with those from a previous NR experiment [39], we used WebPlotDigitizer (Version 4.6) to extract the data from Fig. 3a in [39].

### Statistics

**Fig. 1:**
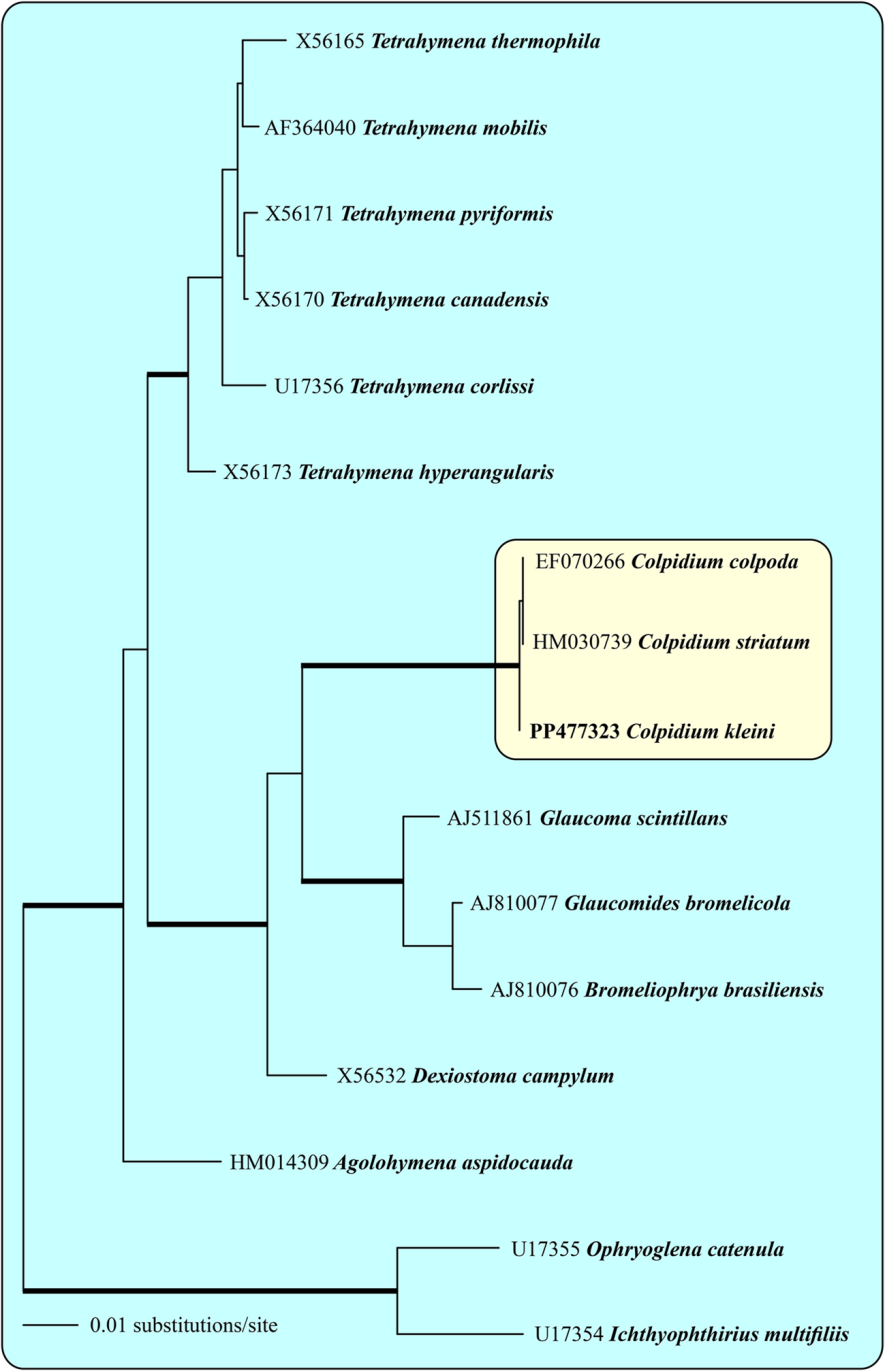
SSU rDNA phylogeny of 16 hymenostomatid taxa inferred from 1737 aligned positions of the sequences analyzed by the maximum likelihood, neighbor joining, and maximum parsimony methods in PAUP 4.0a169. All bootstrap analyses highly support the thick branches (bootstrap values > 75% calculated with PAUP)

Statistical analyses were conducted with the R Statistical Software (v 4.0.5; [40]) if not stated otherwise. Thermal performance curves (TPC) fitted the experimental data (Table 1 and Fig. 1) using the nonlinear Lactin2 model (package rTPC; [41]. This model best fits left-skewed TPCs with a relatively sharp decline at temperatures above *T*_*opt*_ [12].

**Table 1:** Parameter estimates (see Eq. (2) for definition and units), standard errors (SE; for *r*_*max*_, *P' and k*) or 95% confidence intervals (CI, for *δ*_*max*_), and significance levels (*p*) of the numerical response curves shown in Fig. 1. Significant values in bold; *δ*_*max*_ (d^−1^) estimates in parentheses are outliers

T (°C)	Parameter	Value	SE/CI	*p*
**Bacterial abundance** (10^6^ bacterial cells mL^−1^)				
**5**	*r*_*max*_	**0.5487**	**0.1355**	**0.0006**
	*P'*	**0.3307**	**0.1392**	**0.0276**
	*k*	0.8712	0.7080	0.2328
	*δ*_*max*_	-0.34	-1.08	
**10**	*r*_*max*_	0.5091	0.2741	0.0788
	*P'*	**2.2253**	**0.4045**	** < 0.0001**
	*k*	2.9493	1.8471	0.1268
	*δ*_*max*_	(-1.56)	(-6.49)	
**15**	*r*_*max*_	**1.2145**	**0.4366**	**0.0119**
	*P'*	**1.0517**	**0.5382**	0.0656
	*k*	3.3707	3.5695	0.3569
	*δ*_*max*_	-0.55	-2.73	
**19**	*r*_*max*_	**1.0970**	**0.1765**	** < 0.0001**
	*P'*	**0.4782**	**0.1309**	**0.0018**
	*k*	**1.6911**	**0.7900**	**0.0462**
	*δ*_*max*_	-0.43	-1.13	
**21**	*r*_*max*_	**0.6057**	**0.1829**	**0.0037**
	*P'*	**1.3392**	**0.2335**	** < 0.0001**
	*k*	2.6633	1.4792	0.0877
	*δ*_*max*_	-0.61	-1.50	
**Bacterial biomass** (mg C L^−1^)				
**5**	*r*_*max*_	**0.5164**	**0.1243**	**0.0005**
	*P'*	**0.0674**	**0.0293**	**0.0320**
	*k*	0.1583	0.1450	0.2877
	*δ*_*max*_	-0.38	-1.62	
**10**	*r*_*max*_	0.8174	0.8573	0.3523
	*P'*	**0.4273**	**0.0959**	**0.0003**
	*k*	1.1180	1.1314	0.3355
	*δ*_*max*_	-0.51	-1.02	
**15**	*r*_*max*_	**1.1254**	**0.2657**	**0.0004**
	*P'*	0.0498	0.0502	0.3340
	*k*	0.6900	0.4558	0.1465
	*δ*_*max*_	(-0.09)	(-0.35)	
**19**	*r*_*max*_	**1.0223**	**0.1375**	** < 0.0001**
	*P'*	**0.0487**	**0.0089**	** < 0.0001**
	*k*	**0.1094**	**0.0503**	**0.0432**
	*δ*_*max*_	-0.82	-2.43	
**21**	*r*_*max*_	**0.6536**	**0.1931**	**0.0031**
	*P'*	**0.1927**	**0.0275**	** < 0.0001**
	*k*	0.3568	0.1792	0.0611
	*δ*_*max*_	-0.77	-1.92

**Fig. 2:**
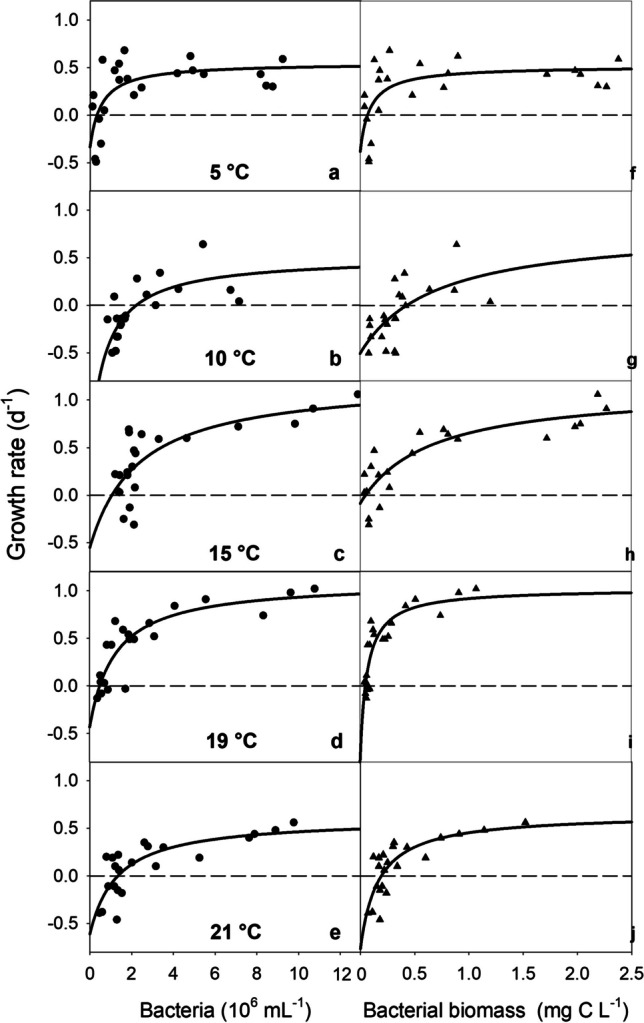
Numerical response of *Colpidium kleini* at temperatures ranging from 5 °C (top) to 21 °C (bottom). Left column: growth rate vs. initial bacterial abundance (dots); right column: growth rate vs. mean bacterial biomass (triangles)

We first used the growth rates from the numerical response curves (Fig. 2a − e) measured at the three to five highest bacterial levels (i.e., at near-to-satiating food levels) at each temperature to fit the thermal performance curve (TPC1) of *Colpidium kleini* (Fig. 2). We then repeated the analysis using the *r*_*max*_ predicted from the NR curves (Table 1; TPC2). We discarded the results from the 10 °C-experiment because the highest bacterial levels in this treatment were lower than in the other treatments (Fig. 2b, g) and the estimated *r*_*max*_ was significant (Table 1).

SigmaPlot for Windows provided test statistics, including error terms for the parameter coefficients of the NR curves.

The response of *δ*_*max*_ to temperature was determined by least-squares linear regression. The slope of the regression was tested for significant deviation from zero (t-test; *t* is the ratio of the regression coefficient to its standard error).

A paired t-test was used to compare the *r*_*max*_ predicted from the NR curves fitted to the initial bacterial abundance and the mean bacterial biomass.

All values reported in this study were considered significant if *p* was < 0.05.

### Morphological Identification

The *Colpidium kleini* strain CIL-4 was identified from living specimens, protargol-stained individuals, and after dry silver nitrate impregnation [42, 43]. The individuals were studied under bright field and differential interference contrast optics with an Olympus BX51 microscope (Olympus, Vienna, Austria) at × 40– × 1000 magnifications. For documentation and measurements, a digital image analysis system was used (Jenoptic PROGRES Gryphax Arktur, Jena, Germany). The taxonomic affiliation of *C. kleini* followed the keys of [24] and [30].

### Molecular Identification and Phylogenetic Analysis

For molecular identification, a single cell of *C. kleini* was washed in several drops of sterile modified WC medium, and the whole genome of this cell was amplified using the REPLI-g Single Cell method (Qiagen, Catalog no. 150343, Hilden, Germany). This approach followed the protocol provided by the manufacturer. The SSU and ITS rDNA were sequenced in a two-step PCR amplification described in detail by Frantal et al. [44]. The SSU gene sequence of *C. kleini* is 1737 bp long with a GC content of 42.2%. The sequences have been deposited in the GenBank database with accession number PP477323.

The assembled SSU/ITS of *C. kleini* was compared using the BLASTn search approach [45] to find the closest relatives. The SSU rDNA sequence was included in a Hymenostomatida dataset, which was aligned according to the secondary structure.

The phylogenetic analyses were conducted using the program PAUP version 4.0a169 [46]. The automated model selection implemented in PAUP was employed to find the evolutionary model that best fitted the dataset. The best model was chosen according to the Akaike Information Criterion ([47]). The setting of the best model was given as follows: GTR + I + G (base frequencies: A 0.2953, C 0.1825, G 0.2489, U 0.2733; rate matrix A-C 0.5430, A-G 3.066, A-U 1.7851, C-G 0.7167, C-U 5.2540, G-U 1.0000) with the proportion of invariable sites (I = 0.7736) and gamma shape parameter (G = 0.5182).

## Results

### Morphological Identification of *Colpidium kleini*

The morphological features of the strain CIL-4 used in the present study matched the description of *Colpidium kleini* by [30] and [24]). Notably, the cortical silverline system is typical for *C. kleini*, i.e., between two ciliary rows (= 1st order meridian), one 2nd order meridian could be observed (Supplementary material, Fig. S1). The cells were kidney-shaped in lateral view. After protargol staining, the cells measured 95 ± 7 × 40 ± 5 µm (*n* = 12; Supplementary Table S1); the ellipsoidal single macronucleus had a size of 22 ± 3 µm (*n* = 11), and one almost globular micronucleus of 2.8 ± 0.2 × 2.1 ± 0.3 µm (*n* = 9) was attached. The contractile vacuole and the excretory pore were located below the cell’s median on the right side of the cell (distance from the anterior cell end to the excretory pore was 57 ± 4 µm, i.e., in a ratio of 1.7 in protargol preparations). The somatic ciliature comprised 39 ± 2 kineties (*n* = 7); the oral ciliature agreed well with *C. kleini*; several elongated caudal cilia were present.

### Molecular Phylogeny of the Genus *Colpidium*

Phylogenetic analyses of SSU rDNA sequences revealed that *Colpidium kleini*, together with *C. striatum* and the type species *C. colpoda*, formed a monophyletic lineage among the Hymenostomatida (Fig. 1). All bootstrap methods highly supported the phylogeny. Notably, the SSU rDNA sequences of the three species differed only in one base position. In addition, only one base difference could be observed in the ITS sequence between *C. kleini* and *C. striatum* (HM030739), supporting their close relationship.

### Experimental Results: Numerical Response and Thermal Performance Curves

The specific growth rates (*r*_*max*_) estimated from the NR curves were 0.52 d^−1^ and 0.55 d^−1^ (based on bacterial abundance, respectively bacterial biomass) at 5 °C, peaked at 15 °C (1.13 − 1.21 d^−1^), were slightly lower at 19 °C (1.02 − 1.10 d^−1^), and decreased sharply at 21 °C (0.61 − 0.65 d^−1^, Table 1). *Colpidium kleini* did not tolerate temperatures ≥ 22 °C. The estimated *r*_*max*_ was significant at all but one temperature (10 °C, *p* = 0.079 and *p* = 0.352, Table 1). The specific growth rates were not different between the NR curves fitted to the initial bacterial abundance (Fig. 2a − e, left column) and the mean bacterial biomass (Fig. 2f − j, right column) (*p* = 0.684).

The threshold levels (*P'*), ranging from 0.3 − 2.2 × 10^6^ bacterial cells mL^−1^, respectively 0.05 − 0.43 mg C L^−1^ (Table 1), did not show any clear trend in response to temperature. However, *P'* was lowest at the lowest temperature tested (at 5 °C, for bacterial abundance) and at 19 °C (for bacterial biomass), i.e., close to the temperature optimum of *C. kleini* (see below). In most cases, the estimates of the constant *k* were not significant (Table 1).

**Fig. 3:**
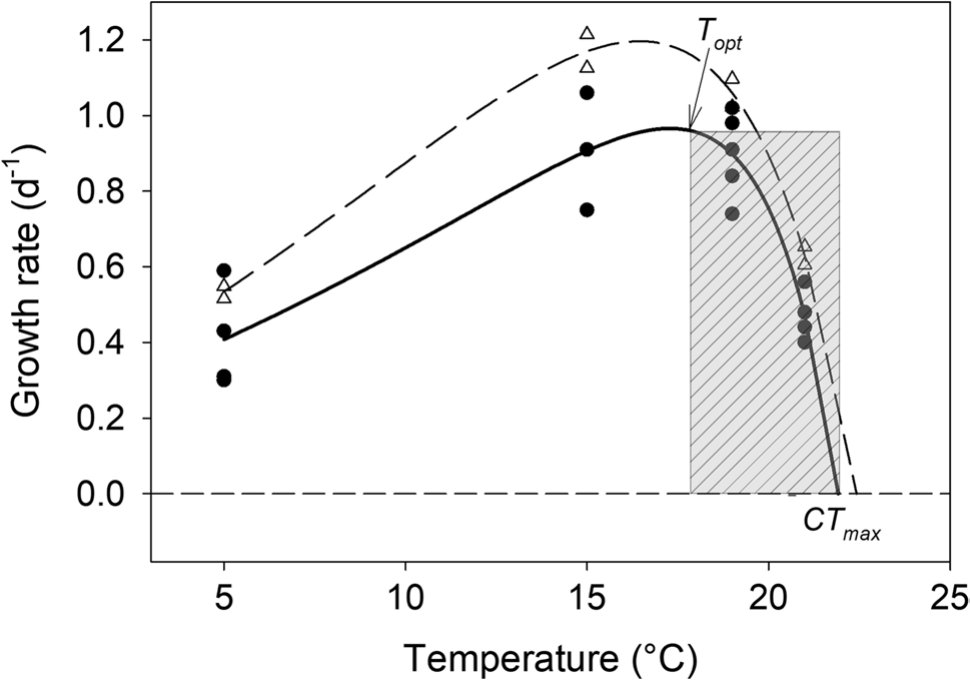
Thermal performance curve of *Colpidium kleini* fitted by the Lactin2 model to the highest growth rates measured (TPC1, see Fig. 2; filled circles and solid line) and the maximum growth rates predicted from the NR curves ( TPC2, *r*_*max*_ reported in Table 1; open symbols and dashed line). The hatched area (gray) indicates the thermal safety margin (TSM = *CT*_*max*_ − *T*_*opt*_) for TPC1

The Lactin-2 model predicted *Colpidium*'s *T*_*opt*_ at 17.3 °C, *CT*_*max*_ at 21.9 °C, and *r*_*max*_ of 0.97 d^−1^ for the measured growth rates (TPC1, solid line in Fig. 3). The thermal safety margin (TSM, i.e., the temperature difference between *CT*_*max*_ and *T*_*opt*_) was 4.6 °C. Using the *r*_*max*_ predicted from the NR curves yielded *T*_*opt*_ at 16.4 °C, *CT*_*max*_ at 22.4 °C, *r*_*max*_ of 1.20 d^−1^, and a TSM of 6.0 °C (TPC2, dashed line in Fig. 3).

The Lactin-2 model yielded significant parameter estimates in both cases. However, the model did not provide a realistic estimate of the lower thermal tolerance limit (*CT*_*min*_) because the steepness of the rising portion of the curve was too low, i.e., the curve asymptotically approached the *x*-axis intercept at temperatures below zero.

**Fig. 4:**
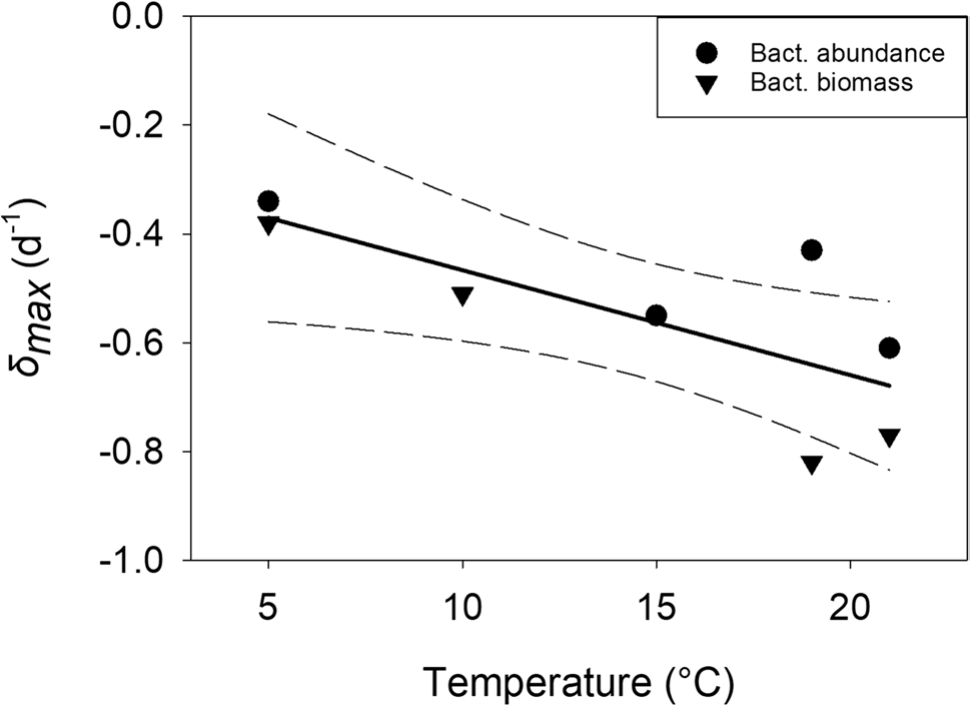
Maximum mortality rates of *Colpidium kleini* vs. experimental temperature. The solid line is the least-squares linear regression; the dashed lines denote the 95% confidence interval. The data were obtained from the NR curves shown in Fig. 2 and are reported in Table 1

The temperature sensitivity of the ciliate’s maximum mortality rate (*δ*_*max*_, d^−1^), estimated from the slope of the least-squares linear regression (Fig. 4), was -0.019 ± 0.007 (SE) °C^−1^. The slope of the regression was significant (*p* = 0.031). Two outliers listed in parentheses in Table 1 were removed from the analysis.

### Cell Size Under Experimental Conditions

The cell size of Lugol’s-fixed *C. kleini*, as measured at the end of the experiments, declined with temperature (Table S1). The specimens measured from the experiments represented the range from starved to well-fed cells. At satiating food conditions, cell size was larger (Table S1). Cell lengths and widths fell into the range previously reported for *C. kleini* [30, 31].

## Discussion

This study presents an integrative approach, combining morphology with molecular species identification and marrying ecology with taxonomy. As a result, we provide significant functional traits related to the species morphology (cell size and shape) and ecology (*r*_*max*_, *P'* [threshold food concentration], *CT*_*max*_, *T*_*opt*_, and TSM). Furthermore, the phylogenetic position is essential for estimating the taxonomic distinctness of a species [1, 48].

We investigated a freshwater ciliate, but our analysis may serve as a model for other taxa, both aquatic and terrestrial. We will critically evaluate our significant findings before discussing this work’s general relevance and implications.

### Identification and Taxonomic Affiliation of *Colpidium kleini*: A Note of Caution

In most cases, no single morphological method can reveal all the characteristics necessary to identify a ciliate species correctly [42]. Therefore, we combined observations of living cells and detailed inspections of protargol-stained individuals. Additionally, we applied a dry silver impregnation to elucidate the details of specific cortex features (i.e., the silverline system). The silverline pattern reveals lines connecting basal bodies of the cilia and other cortical organelles, such as the cytopyge and extrusomes [42]. This analysis is essential to differentiate *Colpidium kleini* from its close relatives *C. colpoda* and *Dexiostoma campylum* (syn. *C. campylum*; see [24] for further discussion). Overall, the morphological characteristics of our strain CIL-4 match well with *C. kleini*.

In our phylogenetic analyses, *C. kleini* formed a monophyletic lineage with *C. striatum* and *C. colpoda*. The relationship among the three strains suggests that they are possible synonyms (Fig. 1; cf. Figure 100 in [49]). Foissner and colleagues [23, 24] already discussed that *C. colpoda* and *C. kleini* were almost indistinguishable from life inspection. They suggested discriminating the two species mainly based on their silverline system, i.e., having two meridians of the 2nd order in *C. colpoda* compared to mostly one meridian of the 2nd order in *C. kleini*. However, even silverline patterns and other features may overlap in both species, and Ganner and Foissner [30] already supposed a “complex of sibling species.” *Colpidium striatum* has also been considered a synonym of *D. campylum* and *C. kleini* [30, 50]. In conclusion, the taxonomic affiliation of the *Colpidium* strain used in this study should be considered with some reverse.

### Feeding Behavior of *Colpidium kleini*

Although *Colpidium kleini* is not a euplanktonic species [24], it occurred in the pelagial of Rifflsee. This lake is characterized by highly abundant particles originating from constant glacial inflow. This particle load causes the high turbidity typical of many alpine lakes [27]. It seems plausible that the ciliate was mainly associated with particle clusters and consumed free-living bacteria and attached bacteria present in the biofilm.

A similar feeding behavior might have occurred in our experiments. We used heat-killed bacteria as food, which likely (partially) sedimented during the experiments (24 h). Some evidence for the assumption that suspension-feeding is not the primary mode of bacterial uptake by *C. kleini* was provided by numerical response experiments with closely related species. Taylor’s experiments [25] with four freshwater bacterivorous ciliates at 20 °C yielded (unrealistically) high threshold levels (4.0 − 25 × 10^6^ cells mL^−1^) for three species (*Colpidium* [syn. *Dexiostoma*] *campylum*, *C. colpoda*, and *Cyclidium glaucoma*) when the ciliates were feeding on suspended bacteria. The fourth ciliate Taylor [25] investigated, *Glaucoma scintillans*, did not feed on suspended bacteria but efficiently grazed on settled *Klebsiella aerogenes* at low bacterial levels. Likewise, *D. campylum* showed a reduced threshold level (3.2 × 10^6^ cells mL^−1^) and higher growth rate when feeding on attached bacteria, indicating that this species may not be a typical suspension feeder [25].

### Functional Ecology of *Colpidium kleini*: Evidence from Numerical Response Experiments

After Taylor’s pioneering work [25] discussed in the previous section, this is the first study reporting reliable parameter estimates from NR experiments for a freshwater bacterivorous ciliate. The only other bacterivorous ciliate for which NR data are available is the marine oligotrich *Strombidium sulcatum* [39]. In their NR experiments, these authors did not report a threshold bacterial concentration with *Pseudomonas* sp. as food. We refitted their data (Fig. 3a in Fenchel and Jonsson [39]) using Eq. 2 and did not obtain a threshold (*P'*) significantly different from 0. However, since *S. sulcatum* grew well (*r* ≈ 0.9 d^−1^) at the lowest bacterial abundance (~ 1 × 10^6^ cells mL^−1^) tested by Fenchel and Jonsson [39], *P'* must have been lower than this prey level. Accordingly, *P'* of this marine ciliate was comparable to the threshold we reported in the present study for the freshwater species *C. kleini* (0.3 − 2.2 × 10^6^ bacterial cells mL^−1^).

Importantly, we measured the lowest bacterial threshold of *C. kleini* at the lowest temperature tested (5 °C). Accordingly, our results matched the bacterial abundance in the cold alpine lake where the ciliate was isolated.

In terms of bacterial biomass, the feeding threshold of *C. kleini* ranged from 0.05 to 0.43 mg C L^−1^, i.e., *P'* falls within the food thresholds known from algivorous and omnivorous marine and freshwater oligotrich and choreotrich ciliates [5, 51, 52].

Several studies, which were not designed as NR experiments, provided cursory evidence that the freshwater species *C. striatum*, a likely synonym of *C. kleini* (discussed above), can grow at or below bacterial densities of 1 × 10^6^ bacterial cells mL^−1^ [53–56]. The specific growth rates of *C. striatum* measured at 15 °C and low bacterial densities [55] were close to our estimates for *C. kleini*.

We postulated that NR curves would overestimate *r*_*max*_ because the rectangular hyperbolic model often predicts *r*_*max*_ at food levels beyond those studied and commonly encountered in situ. Food saturation was not reached in three of the ten cases (Fig. 2 c, g, and h) in our treatments with *C. kleini*. Therefore, the curve fitting predicted *r*_*max*_ higher than the observed growth rates, confirming our hypothesis. However, the predicted *r*_*max*_ may be valid for the species because our experimental bacterial levels were close to the conditions encountered at the sampling site. At the low bacterial density encountered in Rifflsee (0.8 − 1.1 × 10^6^ cells mL^−1^) and low temperature (5 °C), *C. kleini* showed positive growth rates ranging from 0.18 to 0.24 d^−1^ (Fig. 2a). *Colpidium kleini* will meet higher bacterial levels in smaller and more nutrient-rich water bodies that seem to be its typical habitat.

The specific growth rates we obtained for *C. kleini* were much lower than *r*_*max*_ (1.9 − 4.1 d^−1^) of the three *Colpidium* species Taylor [25] investigated at extremely high food levels (4 − 12 × 10^7^ bacteria mL^−1^) and more typical of planktonic ciliates [20, 57]. High growth rates (*r*_*max*_ ≈ 3.0 d^−1^) were also reported for *C. striatum* at 20 °C and bacterial abundances of 2 × 10^7^ bacterial cells mL^−1^ [53]. We conclude that the *C. kleini* strain we used is adapted to the low temperature and bacterial levels typical of Rifflsee. However, the species can survive and should reach higher growth rates in temperate lakes with higher bacterial abundances.

The specific growth rates were not different between the NR curves fitted to the initial bacterial abundance, respectively, the mean bacterial biomass. This finding, confirming our null hypothesis, is vital for comparing *r*_*max*_ across different ciliate taxa and studies. If the respective food levels do not change strongly during the incubation, either method should yield reliable results. However, food must also be supplied at satiating levels to obtain realistic species-specific estimates of *r*_*max*_.

### Mortality Rates of *Colpidium kleini*

The ciliate’s maximum physiological mortality rates, which we calculated from the NR experiments, increased with temperature by 0.019 °C^−1^. This estimate is lower than the average mortality increase with temperature recently reported for three other freshwater ciliates (0.09 °C^−1^, [58]). However, ciliate mortality rates are species-specific and highly variable. The recent meta-analysis of all (77) available ciliate mortality studies (including freshwater and marine taxa) reported that *δ*_*max*_ appears unaffected by temperature [20]. The author concluded that this finding requires more research with contrasting ciliate species [20].

Note that Eq. (2) and the slope of the linear regression in Fig. 4 yielded negative estimates of *δ*_*max*_. For direct comparison with *r*_*max*_, *δ*_*max*_ can be multiplied by − 1 to report positive values ([58]). The mean (0.55 ± 0.17 d^−1^) and median (0.53 ± 0.06 d^−1^) *δ*_*max*_ of *C. kleini* thus calculated were close to the median *δ*_*max*_ (0.55 d^−1^) obtained from 35 planktonic ciliate species that do not encyst [20]. What are the ecological implications of these estimates?

At the typical in situ temperature of Rifflsee (8 °C), the linear regression yields a mortality rate of − 0.43 d^−1^. Assuming a moderate ciliate population size of 1,000 cells L^−1^, Eq. (1) predicts this population would be reduced to 2 cells L^−1^ after 2 weeks without any food. However, some bacterial food is always present in situ. Since *C. kleini* can grow at low bacterial densities, it can survive food-limited conditions without encysting.

The physiological mortality does not account for predator-induced mortality. Grazing by copepods and cladocerans reduces the ciliates’ standing stocks in virtually all aquatic habitats [59]. In Rifflsee, cyclopoid copepods (mainly *Cyclops abyssorum tatricus*) are the sole potential ciliate grazers occurring at very low abundances (~ 0.3 individuals L^−1^, [27]). Assuming the mean clearance rate of freshwater copepods (37 mL ind. d^−1^ at 15 °C, respectively 18 mL ind. d^−1^ at 8 °C [59]) would yield a copepod community grazing rate of 0.005 d^−1^ in Rifflsee. This estimate is ~ 80-fold lower than *δ*_*max*_ and 100-fold lower than *r*_*max*_ (0.52 − 0.55 d^−1^, Table 1), strongly suggesting that grazing loss rates are negligible for the ciliate's population dynamics. At 15 °C, *C. kleini*’*s r*_*max*_ was close to 1.0 d^−1^ at the food levels tested (Fig. 2), i.e., it would require > 25 microcrustaceans L^−1^ to fully control the ciliate’s population size by grazing in temperate lakes.

The role of parasitism, another potential loss factor for ciliates [16, 60], remains virtually unexplored for *C. kleini* and most other aquatic ciliates.

In summary, we conclude that top-down control is too low to control the population dynamics of *C. kleini* during the short ice-free season in the small alpine lakes. Instead, the ciliate is bottom-up controlled by the low temperature and food supply.

### Evidence and Implications from Thermal Performance Curves

Based on the measured growth rates, the TPC yielded a lower specific growth rate for *C. kleini*, a higher estimate of *T*_*opt*_, a lower estimate of *CT*_*max*_, and a narrower TSM than the TPC derived from *r*_*max*_ obtained from the NR curves. Since the *Colpidium kleini* strain we used did not survive at 22 °C, we conclude that the latter curve fitting yielded unrealistic parameter estimates (*CT*_*max*_ at 22.4 °C). Likewise, the narrower ciliate’s thermal safety margin, estimated from the TPC based on the measured growth rates, was closer to the typical TSM (~ 5 °C; [21]) of freshwater ciliates than the TSM predicted from using the *r*_*max*_ of the NR curves.

It is generally inappropriate to extrapolate the results obtained with one particular strain to the species level. However, our estimates of *CT*_*max*_ agree with the findings from field studies; until now, *C. kleini* has not been recorded at in situ temperatures > 21 °C [21].

The experimental results suggest that *C. kleini* may initially benefit from rising water temperatures in Rifflsee and similar cold lakes, provided a sufficient food supply. However, this species will probably disappear from suitable habitats in the long term if temperatures rise unless it can avoid warmer zones. Migrating to the more profound and cooler depths close to the lake bottom is an option for bacterivorous species such as *C. kleini* but not for mixotrophic and algivorous ciliates limited by light availability. In the alpine lakes, dwelling in deeper zones is also a successful strategy to avoid UV damage [28, 61]. However, escaping supraoptimal temperatures by downward migration is impossible for *C. kleini* in its typical habitats, such as shallow lakes and ponds. Because *Colpidium* species, other than the closely related genus *Colpoda*, cannot form any kind of cysts ([30]; see also [62]), *C. kleini* is under threat in small, shallow water bodies if lake temperatures further rise.

## Conclusions

We conclude that TPCs are better suited than NR curves to estimate *r*_*max*_ and *T*_*opt*_ over meaningful food ranges, i.e., at prey levels occurring in situ. In contrast, NR curve fitting using Eq. (2) is the better option to predict *r*_*max*_ if food saturation was not reached in the experiments. Accordingly, combining both methods provides two important functional traits of ciliates and other organisms, i.e., the species’ temperature optimum (based on TPCs) and their specific growth rate.

Because Eq. (2) contains an x-axis intercept (*P'*), the constant *k* is not equivalent to the half-saturation constant of the Michaelis–Menten kinetics. However, *k* is a helpful parameter for crudely estimating the food level at which satiation is likely. If all parameters of the NR curve, including *k,* are significant (which was not the case in our study, Table 1), the prey concentration yielding *r*_*max*_ can be calculated accurately from Eq. (2). Such estimates may guide future studies with other taxa, both aquatic and terrestrial.

Specific growth rates estimated from NR curves fitted to the initial bacterial abundance or the mean bacterial biomass yielded similar results. This will likely not be the case if the experimental duration is prolonged to more than 24 h and food levels change strongly during incubation.

Intraspecific morphological and physiological variability are widespread among aquatic ciliates. Furthermore, morphologically indistinguishable species may be genetically different (sibling species). Therefore, the taxonomic identity of the species should be verified unequivocally in experimental ciliate research.

The present study adds to the increasing evidence that many freshwater ciliates are at risk of local extinction if lake warming continues. Like ~ 70 other freshwater ciliates [21], *Colpidium kleini* is a temperature-sensitive species that cannot tolerate temperatures > 22 °C. This ciliate may serve as an indicator species for critical warming in small water bodies.

Supplementary Information.

### Supplementary Information

Below is the link to the electronic supplementary material.

Supplementary file1 (DOCX 1195 KB)

## Data Availability

All data generated or analyzed in this study are included in this article and the supplementary material.
