# Supplementary material for: Numerical and Thermal Response of the Bacterivorous Ciliate Colpidium kleini, a Species Potentially at Risk of Extinction by Rising Water Temperatures
Source: Microb Ecol. 2024 Jul 2;87(1):89. doi: 10.1007/s00248-024-02406-y (PMC11219425; doi:10.1007/s00248-024-02406-y)

**Supplementary material to**

**Numerical and thermal response of the bacterivorous ciliate *Colpidium kleini*, a species potentially at risk of extinction by rising water temperatures**

Thomas Weisse ^a,^*, Thomas Pröschold ^a^, Barbara Kammerlander ^b, c^, Bettina Sonntag ^a^ , Laura Schicker ^a^

## ^a^ Research Department for Limnology, Mondsee, University of Innsbruck, Mondsee, Austria

- ***Corresponding author****; E-mail address:* [thomas.weisse@uibk.ac.at](mailto:thomas.weisse@uibk.ac.at) (T. Weisse).

**Supplementary Table S1**. Cell size of *Colpidium kleini* (mean values + standard deviation; length and width in µm, volume in µm³).

| **T (°C)** | **Length** | **Width** | **Volume** | **n** | **Food level** | **Fixative** |
| --- | --- | --- | --- | --- | --- | --- |
| 10 | **80.3** + 7.7 | **31.4** + 6.3 | **43,274** + 17,557 | 20 | Mixed | Lugol’s iodine |
| 15 | **76.6** + 14.1 | **36.1** + 7.2 | **33,159** + 23,955 | 25 | Mixed | Lugol’s iodine |
| 19 | **68.0** + 13.2 | **27.7** + 3.7 | **28,889** + 12,036 | 15 | Mixed | Lugol’s iodine |
| 15 | **89.9** + 23.3 | **43.0** + 11.7 | **103,318** + 65,024 | 20 | Satiating | Lugol’s iodine |
| 10 | **95.3** ± 6.8 | **39.5** ± 5.2 | **82,297** + 36,561 | 12 | Satiating | Bouin’s solution |


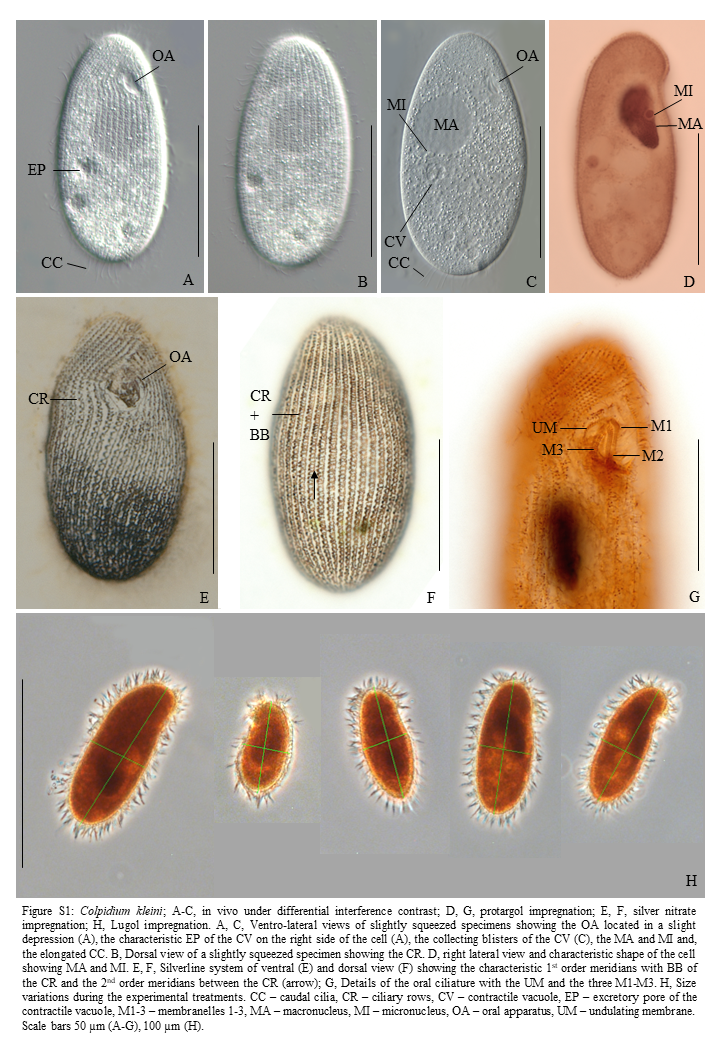

Supplement: Supplementary file 1 — Supplementary file1 (DOCX 1195 KB) [file 248_2024_2406_MOESM1_ESM.docx]
